# Exploring linkages between transport and disaster risk reduction in South Africa: A review of literature

**DOI:** 10.4102/jamba.v11i2.724

**Published:** 2019-07-04

**Authors:** James Chakwizira

**Affiliations:** 1 Department of Urban and Regional Planning, School of Environmental Sciences, University of Venda, Thohoyandou, South Africa

**Keywords:** Transport, Disaster, Risk, Reduction, Framework, South Africa

## Abstract

Transport systems, network densities, design capacities and constraints (including levels of service expressed in terms of quantity and quality) are central to disaster risk logistics, mitigation and adaptation. Using a desktop literature review method, this study analysed headline disaster risk issues in the transport sector of South Africa. The analysis indicated that implementation gaps exist in terms of the operating policy, institutional and legislative framework. The gaps were located at different spheres of government and expressed themselves at different scales. The end result of the disjuncture was a compromised disaster risk reduction service delivery environment. Although existing platforms constitute a good starting point for tackling disaster risk in the transport sector, the article argues that this is not enough. A transport and disaster risk reduction atlas and implementation roadmap are advanced as one way of contributing towards a better transport and risk reduction agenda in South Africa.

## Introduction

Linking transport and disaster risk reduction (DRR) in South Africa is critical as part of the sustainable development goals (SDGs), National Development Plan (NDP 2030) as well as the continued quest to encourage growth and development in the economy. Anecdotal and few studies exist that explicitly link transport and disaster risk science in South Africa. Inadvertently, the existing studies approach transport and disaster risk by default and implication, hence the need to investigate and explore deeply the linkages between transport and disaster risk science.

Exploring the links between transport and DRR in South Africa requires that conceptual definitions of what constitutes DRR, disaster risk management (DRM) and hazards be outlined. This is necessary to overcome the challenge of using the three terms ‘inter-changeably’ and a failure to separate the terms, which creates challenges in various spheres and scales of planning. The International Strategy for Disaster Reduction (UNISDR 2002:25, 2009) defines DRR as:

[*T*]he systematic development and application of policies, strategies and practises to minimise vulnerabilities and disaster risks throughout a society, to avoid (prevent) or to limit (mitigate and prepare) adverse impacts of hazards, within the broader context of sustainable development. (p. 10)

On the other hand, DRM in South Africa, according to Section 1 of the *Disaster Management Act* (DMA), 57 of 2002 (Republic of South Africa 2003), is defined as ‘a continuous and integrated multi-sectoral and multi-disciplinary process of planning and implementation of measures aimed at disaster prevention, mitigation, preparedness, response, recovery, and rehabilitation’ (Vermaak & Van Niekerk 2004:556). In this regard, DRM seeks to reduce the vulnerability of communities at risk through improved access to services, development opportunities, information, education and empowerment (Van Zyl 2015:18). An understanding of DRR and DRM in the absence of a conceptual clarity regarding the concept of a hazard is incomplete. According to Niekerk (2002), hazards can be single, sequential or combined in their origin and effects. Each hazard is characterised by its location, intensity and probability. Typical examples of hazards can be the absence of rain (leading to drought) or the abundance thereof (leading to flooding).

Closely linked and connected with the DRR concept, hazards as well as the DRM concepts are the concepts of vulnerability. Vulnerability as a concept is related to the ‘degree to which an individual, a household, a community or an area may be adversely affected by a disaster’ (Van Niekerk 2006:97; Republic of South Africa 2003). Vulnerability is best understood when co-considered with the concept of resilience. Resilience is a measure of the ‘capacity to absorb and recover from the impact of a hazardous event’ (Van Zyl 2015:18). Within the transportation sector, transport presents a number of potential disasters such as accidents, gas and fuel explosion (i.e. including fire hazards), congestion, emissions that have both global and localised impacts, and effects such as health impacts owing to respiratory diseases. Transport is, therefore, both a disaster risk domain as well as a catalyst and enabler in disaster risk responses, mitigation and adaptation (i.e. with respect to logistics distribution affecting service delivery in terms of response times and delay times) (World Bank 2010:25, 2013:6; Gauteng Bulletin 2012:4). This article adopts a textbook definition of a transportation system or mode as a ‘system(s) for moving persons or goods’ and is entirely made up of three components, namely vehicles and equipment, guideway and/or carriageway and operational plan as advocated by Boyce (2009:1).

Table 1 reveals that the impacts on the built environment and ecosystems are significant. However, we should not lose sight of the impact of natural hazards and climate change. While natural disasters have well-known effects such as the immediate destruction of infrastructure and loss of lives, climate change and other natural hazards have less obvious, incremental impacts on human settlements, communities and residents.

**TABLE 1:** Incremental impacts of climate change and natural hazards on human settlements, communities and residents.

Incremental impacts on human settlements and built environments	Impacts on communities and residents	Natural environment
Stress on building foundations Road washouts Changing disease vectors Stress on stormwater and sewage systems Stress on water treatment systems Disruption to shipping and ports Increased energy demand Increased road surface damage Increased demand for water	Illnesses: heat stress, stroke, malnutrition, water-borne diseases, asthma, physical and mental disability Exposure to elements from substandard construction Disruption of basic service provision and access to supplies Housing instability Property loss and relocation Loss of livelihoods Community fragmentation Exposure to flood-related toxins and wastes Disruption in availability of potable water, food and other supplies Water shortages Food shortages; Higher food prices Disruptions of electricity	Coastal erosion, altered ecosystems and wetlands Salinisation of water sources Slope instability Groundwater depletion Reduction in greenspace and growing conditions including urban agriculture Changes in fish populations Increased runoff contamination Increase heat island effect Increased air pollution

*Source*: Adapted from World Bank (2011:4)

Table 1 clearly explains why disaster risk in the transport sector is widely understood as a function of hazards and vulnerability of livelihoods and economies (UNISDR 2009:9–10).

### Objectives or research questions

The main objective of the article is to illustrate the links between transport and DRR in South Africa. This objective is achieved through answering the following three questions:

1. What are the major transportation and DRR issues facing contemporary South Africa?
2. Which policy, legal and institutional frameworks exist to facilitate integrated transport and DRR in South Africa and why?
3. What are a suite of potential interventions and initiatives to strengthen the DRR component in the transportation sector?

## Methodology

The research is based on an extensive desktop survey of existing literature. The qualitative methodology adopted uses a thematic approach to identifying issues and analysing the major transport and disaster risk issues. In addition, the gap analysis technique is used in confirming transport and disaster risk challenges requiring attention. Selected key stakeholders and institutions were purposively sampled to solicit their views and opinions on the adequacy or inadequacy of existing and proposed transport and disaster risk plans, policies and/or programmes.

### Literature review

In 2009, South African President Jacob Zuma pledged to the United Nations Framework Convention on Climate Change (UNFCCC) to reduce emissions by 34% by 2020 and 42% by 2025 relative to business as usual and contingent on international support (Boyd, Coetzee & Boulle 2014:1). Central to achieving success in reaching these stated targets is the need for the transport sector to undergo a transition towards a low carbon economy among other initiatives. This section reviews the key literature that links transport and DRR in South Africa.

#### Disaster risk reduction, climate change and transport sector adaptation

Climate change, DRR and transportation interact in fundamentally two distinct but complementary ways. At the first level, it has been established that short-term climate variability influences the frequency and range of shocks that impact on communities and societies (IPCC 2007:7; United Nations 2013). In this scenario, the transportation sector acts as a fulcrum that facilitates and bridges the employment and development of responsive mitigation and adaptation platforms that either absorb or adjust to such induced system shocks. On the other hand, in the long run, climate variability can give rise to changes in the productive base of communities and societies (Schipper 2009; Schipper & Pelling 2006:19-38). This is more acute for those communities and societies that are ‘resource dependent’ with implications for building, construction and the deployment of transportation resilient infrastructure and services. Secondly, climate change is changing the ‘frequency, intensity, spatial extent, duration, and timing of extreme weather and climate events, and can result in unprecedented extreme weather and climate events’ (Shamsuddoha et al. 2013:9). Both disasters irrespective of whether they are climate-induced or otherwise created impact negatively in terms of affecting development outcomes in space and time (IPCC 2007:5).

#### Disaster risk reduction and transportation overview in South Africa

Efficient access and mobility movement are at the centre of economic competitiveness of both urban and rural economies in any place. It is for this reason that one may argue that the level of investment and quality of infrastructure are critical for enhanced socio-economic growth and development of any region, area or country. Measured against its southern African counterparts from a transportation infrastructure provision, South Africa has a comparatively well-developed transportation infrastructure network (National Treasury 2011:161).

However, the challenge is that despite this highly developed transportation network system, South Africa has missed a generation of capital investment in roads, rail, ports, electricity, water and sanitation, public transport and housing dating from the 1970s (Department of Transport 2013). The current infrastructure allocations relative to gross domestic product (GDP) are about 7%, which has been identified as being inadequate (Department of Transport 2013:4). In the short term, rail and rural road infrastructure in particular has been neglected and under-maintained. This is in part because of the transport industry competing for funding from the fiscus with other government or public sector services and funding being allocated to key priority areas (Department of Transport 2015:28). The NDP (2030) calls for a gross fixed capital formation of about 30% of GDP by 2030 to see a sustained impact on growth and household services (NDP 2011). The NDP (2030) calls for effective, reliable, economical and smooth-flowing transport corridors. Roads and railways are intended to be facilitators of connectivity and mobility. However, roads in particular have increasingly become bottlenecks because of congestion and pavement damage by excess axle loads of trucks among other considerations (Department of Transport 2015:27). The South African road network has a total length of 750 000 km, of which an estimated 17.6% comprises public roads that are not formally gazetted by the authority (i.e. unproclaimed gravel roads) (SANRAL 2014). Of the proclaimed road network, 25.5% is paved and 74.5% is gravel roads (Department of Transport 2015:27).

The road network is characterised by wide disparity in condition, among the different categories, which has implications regarding DRR in the event of transportation hazards. In 2013, SANRAL recorded that 26% of municipal and provincial roads were in a poor to very poor condition and 38% were in a good to very good condition (SANRAL 2014). The management and maintenance of roads is relatively average in provincial and municipal departments although the South African National Roads Agency Limited (SANRAL) operations meet or exceed world standards with 48% of the SANRAL network in good to very good condition (SANRAL 2014). Although national roads exceed world standards in South Africa, however, close to 80% of the national road network has exceeded its 20-year structure lifespan and as such highlights the critical need for effective and appropriate maintenance. This also resonates with DRR regarding the vulnerability and resilience of existing transportation infrastructure in the event of a hazard as aged infrastructure damage likelihood is high.

**FIGURE 1:**
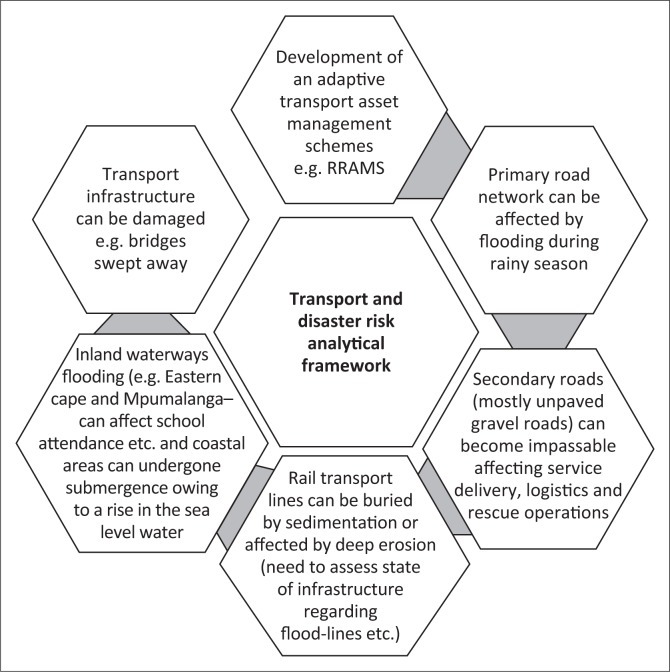
Transport and disaster risk analytical framework in South Africa.

Racially segregated town-planning outcomes of the apartheid era, along with rapid urbanisation, and urban sprawl continue to plague South Africans with long and costly travel distances, particularly for the urban poor, to access economic opportunities and social services. This has also implications regarding disaster risk exposure, vulnerabilities and challenges for the urban and rural poor in South Africa. Overall, although the transportation sector has a host of progressive legislation and policy frameworks, it is constrained in terms of optimised DRR in the event of a hazard or disaster by a combination of infrastructure backlogs or deficits, skills gaps and shortages as well as funding gaps and deficiencies that can be traced to having started occurring in the 1970s. Figure 1 shows the transportation challenges and by extension opportunities for tackling DRR constraints in South Africa. In reality, these identified hazards that are not exhaustive but only illustrative act in complex and integrated ways making tackling transportation and DRR matters a challenging activity.

The South African transportation sector is highly dependent on petroleum and its derivatives. Petroleum is the single largest import item, representing about 80% of the country’s primary energy imports and in the process using the majority of the earnings from mineral exports (BalticBiogasBus 2012:4). As such, South Africa is acutely vulnerable to scarcity of oil which is a non-renewable resource. In this regard, ‘identifying alternative sources of energy is a matter of urgency’ (Greben, Burke & Szewczuk 2009:1). The need to explore alternative energy sector to support the transportation sector is perhaps one reason why interventions to support innovation can be argued to be both necessary and justifiable.

Generally, South Africa is committed to participating in the mitigation of global climate change and also has a direct interest in addressing the ill effects of transport (Das & Keetse 2015:835). These ill effects include noise pollution, poor air quality (particularly from particulates) and congestion. South African cities are becoming highly congested with traffic, for example, with over 700 vehicles per kilometre. Gauteng Province has the highest road density in South Africa being accommodated on the smallest road network (National Treasury 2009). Congestion increases emissions, decreases the liveability of cities and represents a major economic cost. Poor air quality also plagues South Africa, and urban air quality is a major risk factor for death (Suleman, Gaylard, Tshaka & Snyman 2015:12–13). Addressing transportation challenges is a crucial lever in generating a transportation compliant DRR framework that is robust, flexible, resilient and sustainable for all transportation modes in South Africa.

## Research analysis and findings

This section presents firstly the existing policy, legal and institutional frameworks in support of DRR and integration with the transportation sector. Secondly, the transport and DRR opportunities and initiatives in South Africa are discussed. Thirdly, a conceptual framework for integrating DRR in South Africa is outlined.

### Policy, legal and institutional frameworks exist to facilitate integrated transport and disaster risk reduction in South Africa

**FIGURE 2:**
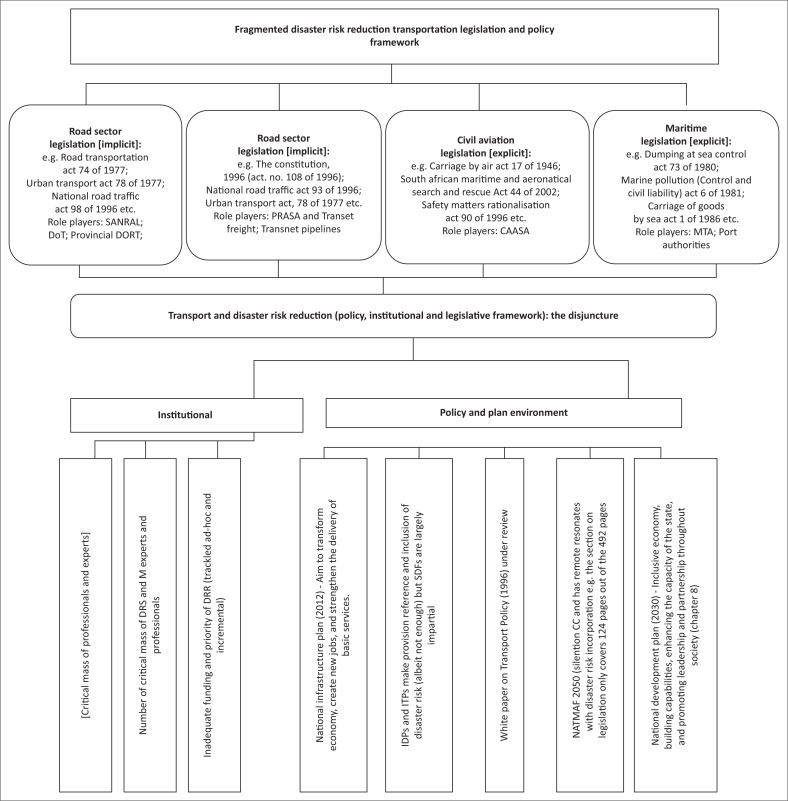
Transportation sector policy, legal and institutional frameworks for disaster risk reduction in South Africa.

South Africa has developed a string of progressive policy frameworks that lay a solid foundation for the implementation of resilient DRR plans, actions and programmes at different scales as well as covering different sectors of the economy. The National Disaster Management Framework of 2005 guides the implementation of the 2002 *Disaster Management Act*, which established the Disaster Management Centre (i.e. the principal unit for national DRM). At the same time, the NDP (2030) mentions disaster preparedness as one of its objectives (NDP 2011). In the line, the National Climate Change Response White Paper highlights the need for additional funding for DRR in the context of climate change (Department of Environmental Affairs 2011:43). Figure 2 shows the policy, legal and institutional frameworks for DRR with particular application to the transportation sector.

As shown in Figure 2, we can deduce that despite the existence of a good policy and legislative environment in the transportation sector to on-ramp the DRR activities, gaps exist regarding the lack of a single transport authority to co-ordinate the transition to a low-carbon economy, especially in the context of local municipalities in various provinces. Each transportation sub-sector is responsible for crafting its own DRR transportation roadmap, which creates challenges of planning, implementation and funding fragmentation and coordination within and beyond the transportation sub-sectors. The need to realign and streamline the DRR component within the broader transportation sector is an aspect that requires attention if mismatches and the disjuncture in implementing DRR measures are to be addressed. What also stands out is that by applying the core principles of assessment, prevention, mitigation and preparedness as part of DRR measures, as advocated in *The Disaster Management Act, 2002*, and the Key Performance Areas and Enablers of the National Disaster Risk Management Framework, many disasters in the transportation sector can either be reduced or prevented (Van Zyl 2015:20). Furthermore, transportation commenters have highlighted that South Africa’s transport sector needs to be altered significantly, to align with the country’s climate objectives. This is because the National Climate Change Response White Paper outlines a Transport Long-term Priority Flagship Programme. In this flagship programme, actions and measures aimed at reducing disasters and hazards from vehicle emissions are presented. These include the development of the Gautrain and MyCiti and Rea Vaya BRT systems in Cape Town and Johannesburg as indicators of progress towards green transport systems transition (Boyd et al. 2014:10). In addition, a host of other initiatives such as the zero emission vehicle pilot programme being overseen by the Department of Trade and Industry (DTI) and Department of Environmental Affairs (DEA) are ways of improving the energy efficiency in the transportation sector (National Treasury 2013:65–66; Greve 2013). Overall, the importance of embedding or integrating DRR into NDP’s priorities is increasingly being seen as the way forward.

### Transport and disaster risk reduction opportunities and initiatives in South Africa

**FIGURE 3:**
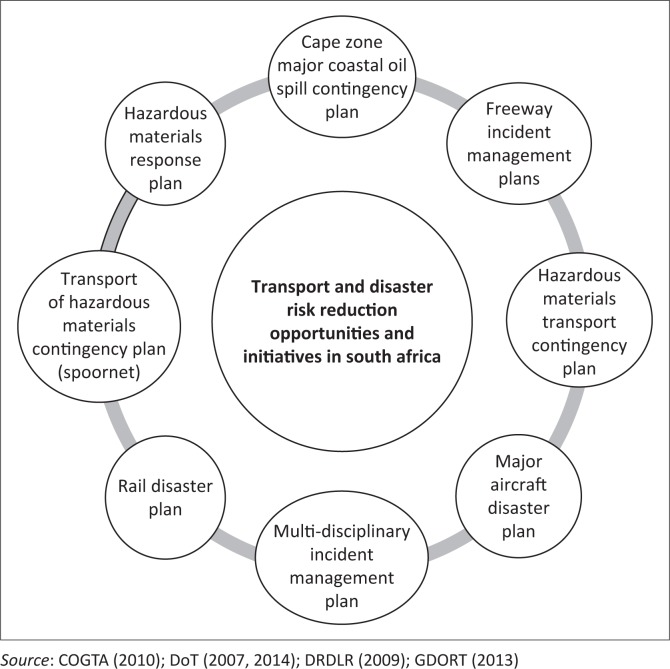
Transport and disaster risk reduction opportunities and initiatives in South Africa.

Although the transportation sector in South Africa is confronted with a host of challenges just like any other country in the world, this section uses challenges as a departure point in generating DRR compliant transportation actions and solutions. Figure 3 shows the transport and DRR opportunities and initiatives in South Africa. The bottom line is that a good base to incorporate and strengthen the DRR component in the transportation sector exists. Although the actual reduction of DRR will be subject to complex and contextual realities for each transportation sub-sector, the general trends and patterns remain stable.

As shown in Figure 3, we can deduce that opportunities and constraints exist regarding DRR in a number of initiatives given as follows:

1. ***Cape Zone Major Coastal Spill Contingency Plan:*** The national contingency plan is currently under revision and extension to include spills of chemicals. Twenty-five Local Coastal Oil Spill Contingency Plans, compiled by the Department of Environment, Agriculture and Tourism (DEAT), detail appropriate actions to be taken upon threatened or actual impact. These are supported by a comprehensive Coastal Sensitivity Atlas. Individual plans for oil-handling facilities are in place, drawn up by the oil industry, represented by the Oil Industry Environment Committee (OIEC). The clean-up of oil spills in port has been delegated to South African Port Operations (SAPO), the ports administration. Plans are established for each port.
2. ***Hazardous Materials Response Plan:*** A substance may be considered hazardous if it is flammable, explosive, toxic, corrosive, radioactive and cryogenic or readily decomposes to give off oxygen at elevated temperatures. There are thousands of substances that possess one or more of these qualities and can therefore be considered as hazardous. Multiple hazards can be associated with many substances, and the intermixing of chemicals can further complicate the behaviour and hazardousness of a substance. Commonly encountered flammable liquids include petrol, oil, diesel, paraffin, benzene, alcohols, pesticides and jet fuel (Burke 2003). These include hazardous material (HAZMAT) risk and atlas plan by key transport route corridors.
3. ***Transport of Hazardous Materials Contingency Plan (Spoornet):*** TRANSNET, DEAT and Department of Water Affairs and Forestry have established formal management systems to monitor and enforce waste management and pollution control measures, in particular along the main transport routes and in areas where hazardous material and waste are likely to be located and or generated. The systems seek to ensure that provincial and local authorities in these areas have sufficient resources to deal with these disasters.
4. ***Rail Disaster Plan:*** In the case of rail accidents involving passenger or goods train, emergency warnings are communicated through the rail company or through the Joint Operating Office of Spoornet which then alert the necessary parties for support. The local authority and relevant national departments and private contractors can be called in to assist in cases where collisions involve hazardous chemicals or substances.
5. ***Multidisciplinary Incident Plan:*** In this case, it is critical that disciplines, sectors, departments and institutions from both the private, public and civil society join hands in addressing, managing and implementing sustainable transport risk reduction and adaptation solutions.
6. ***Major Aircraft Disaster Plan:*** In the case of aviation accidents, an incident is reported directly to the Commissioner of Civil Aviation where the emergency service units at airports or from local authorities should be called in to attend to the disaster. If it is within the airport, it is dealt with by the Airport Company, and if it is outside, it is dealt by the local authority. The Commissioner of Civil Aviation can also call upon the support of the South African Police Services and the South African Search and Rescue (SASAR).
7. ***Hazardous Materials Contingency Plan:*** This requires every transportation plan to have a contingency plan regarding how hazardous materials disaster can be averted, managed and responded to as and when they occur.

### Public and private sector initiatives to build capacity at local government level

A key aspect that was raised by key institutions and stakeholders was the need to build a critical mass of disaster risk professionals, experts, technologists and practitioners to assist with facilitating the development of disaster proof sectors of the economy, including the transportation sector. Table 2 shows public and private sector initiatives to build capacity at local level in South Africa. From Table 2, we can see that a number of initiatives are already in place aimed at promoting capacity building and training of professionals in different fields for enhanced delivery. However, there is no dedicated DRR capacity building and training programme or transportation curriculum, which is a gap that requires to be filled.

**TABLE 2:** Public and private sector initiatives to build capacity at local government level.

Initiative	Purpose
*Project Consolidate Public Initiative:* Launched October 2004	Hands-on assistance to 139 targeted municipalities by experts, dubbed service delivery facilitators (SDFs), with a range of managerial, technical and financial skills.
*Joint Initiative on Priority Skills Acquisition (JIPSA):* Public Initiative with private role players, launched 27 March 2006	To create short-term, sustainable interventions to the skills problems, that is, engineering, planning, artisanal, technical and project management skills at municipal level.
*Siyenza Manje:* Launched jointly by several State Departments, the South African Local Government Association (SALGA) and the Development Bank of South Africa (DBSA)	Re-employment of retired personnel with skills in engineering, project and financial management, and town planning, who would then be assigned to targeted municipalities. The first batch, 67 in total, was recruited between May and November 2006. They provided both hands-on intervention and mentoring to young graduates.
*Skills Importation Public Initiative*	Departments of Labour and Home Affairs were assigned to make acquisition of work permits and entry into the country easier for this category of individuals. This would involve encouraging South African citizens with the relevant skills, who are either working or resident abroad, to return home for employment in the municipal sector.
*Unemployed/Inexperienced Graduates:* Placements Public Initiative launched in 2005	To draw unemployed graduates into the municipal sector, especially in the Departments of Arts and Culture, Environmental Affairs and Tourism, and Public Works. Whilst providing the much needed expertise, albeit inexperienced, to the municipal sector, this measure went a long way to reduce the rate of unemployment among graduates, especially within the black community.
*Public–Private Sector Initiatives:* Training programmes	Private and public institutions undertook joint initiatives to provide training to municipal employees. (e.g. the Old Mutual Business School, assisted by the South African Management Development Institute and the Department of Provincial and Local Government, where 97 municipal employees were provided with hands-on training in foundational project management).
*Municipal Demarcation Board:* Annual capacity assessment of municipalities	Monitoring and Tracking to improve the capacity of municipalities to implement budgets and their service delivery mandate, to measure their ability to meet their obligations in performing their powers and functions.
Mu*nicipal Infrastructure Support Programme:* Agency created 2011	To support municipalities with planning, management and other technical expertise to roll out infrastructure more efficiently and effectively – especially in weaker municipalities. MISA is currently providing technical capacity support to 107 municipalities, with a total of 77 technical experts (engineering and planning professionals) assigned to support these municipalities.
*Platform for Spatial Temporal:* Evidence for planning in SA	Funded by DST and developed as a collaboration between CSIR, HSRC and a range of national, provincial and local role players to provide decision-makers, officials and researchers with access to advanced spatial analyses, data sets and tools to support research, policy making and municipal or regional spatial planning in Urban Growth as well as Regional Development areas.
*Geospatial Analyses Platform (GAP) and CSIR/SACN Functional Settlement Typology*	The result of major policy initiatives and technology developments, the geo-spatial platform facilitates for the assembly, analysis and sharing of economic, development and demand information is used by a wide range of municipalities as basis for spatial development frameworks. The platform enables spatially and temporal specific analyses of settlements and regions, used to inform the National Disaster Unit as basis for National Disaster Management System, the National Development Plan (2012) and the Integrated Urban Development Framework (2014).

As shown in Table 2, we can deduce that initiatives and efforts aimed at improving approaches, techniques, decision-making as well as skilling professionals exist almost for all sectors of the economy. What is perhaps missing and key is to provide for coordinated learning and sharing of the best practices for everyone’s benefit. Establishing a DRR network of practitioners is one way of seeking to tackle this matter.

### Transportation disaster risk reduction framework of analysis

**FIGURE 4:**
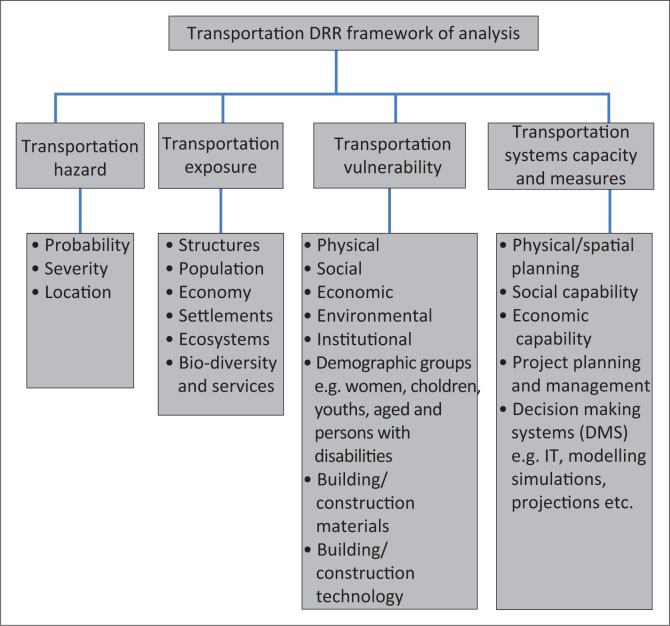
Transportation disaster risk reduction framework of analysis.

Taking the proceeding sections into account, this subsection presents a transportation DRR framework of analysis for use in the various transportation sub-sectors. Although Figure 4 shows a simplified illustration of the framework of analysis, in practice engaging in the processes that are interrelated, interdependent is a complex system approach. However, the overarching principles that should guide the analytical framework are the need to develop resilient transportation infrastructure, services and systems.

Figure 4 shows that the framework of analysis can be applied at different scales from micro-, meso- to macrolevel. In addition, the framework can be applied across various sectors of the economy as well as spheres of government. The framework is based on the assumption that funding for DRR is budgeted and provided for, the existing DRR policy frameworks are authentic and the existing DRR transportation professionals are in posts at various levels within institutions and organisations throughout South Africa.

## Research contribution

The study has established that strengthening the DRR component in the transportation sector requires action and co-ordinated and connected measures from different spheres of government and sectors of the economy. The role that collaboration and partnership at all scales from local, municipal, provincial to national play cannot be over-emphasised. Figure 2 shows the key success factors for linking better transport and risk reduction in South Africa.

As shown in Figure 2, we can deduce that generating and rolling out a sustainable transportation DRR strategy and programme is not an automatic process. There is need for joint or one governance approach. In addition, capacity building and training of transportation cadres with competencies and skills in DRR are important. This resonates with the need to develop customised and relevant curricula, especially for continuous professional development, for professionals such as engineers, surveyors, architects and town planners. Both industry and training institutions have roles to play in ensuring that the required skills are available to respond to the needs of the society.

## Research limitations

This article explores the links between transport and DRR in South Africa; however, the approach is generic and provides a frame for responding to transportation risks in general. The approach is therefore inadequate regarding how each sub-sector in the transport sector can respond to disasters in South Africa, which is a focus of a different study and further research. Investigating specific transportation logistics and risks mapping will lead to the generation of different risk atlases for different transport modes. Exploring the scope to pilot test and implement demonstration projects and programmes aimed at promoting more resilient transportation systems by mode and area is an exciting prospect for research, innovation and dissemination of best practice. In addition, the study did not consider the DRR dimensions for ecosystems, neither was an ecological approach used in seeking to understand transport and DRR in South Africa. These are additional areas for further research.

### Ethical considerations

This article followed all ethical standards for a research without direct contact with human or animal subjects.

## Discussion and concluding remarks

Overall, DRR practices need to be multi-hazard-based, inclusive and accessible to be efficient and effective. Effective transportation and DRR measures and actions must be developed to be resilient and inclusive on the understanding that full-cycle engagement of all stakeholders is non-negotiable. This is in terms of providing space and adequate opportunities for enhanced participation of women, children and youth, persons with disabilities, indigenous peoples, volunteers, the community of practitioners and older persons in the design and implementation of transportation policies, plans and standards. This way constructed and deployed transportation infrastructure and services will be amenable to DRR in the event of hazards and disasters, as existing response plans will cater for the needs of different groups in the society. Consequently, there is a need for the public and private sectors within and beyond the transportation sector to work more closely together and create opportunities for collaboration, innovation and for business to integrate disaster risk into their management practices, investments and accounting systems (United Nations General Assembly [UNGA] 2014:4). Developing a clear transportation and DRR implementation roadmap is central to better and advanced response outcomes for the future in South Africa.
